# Supplementary figures and images for: Functional hierarchies in brain dynamics characterized by signal reversibility in ferret cortex
Source: PLoS Comput Biol. 2024 Jan 19;20(1):e1011818. doi: 10.1371/journal.pcbi.1011818 (PMC10836715; doi:10.1371/journal.pcbi.1011818)

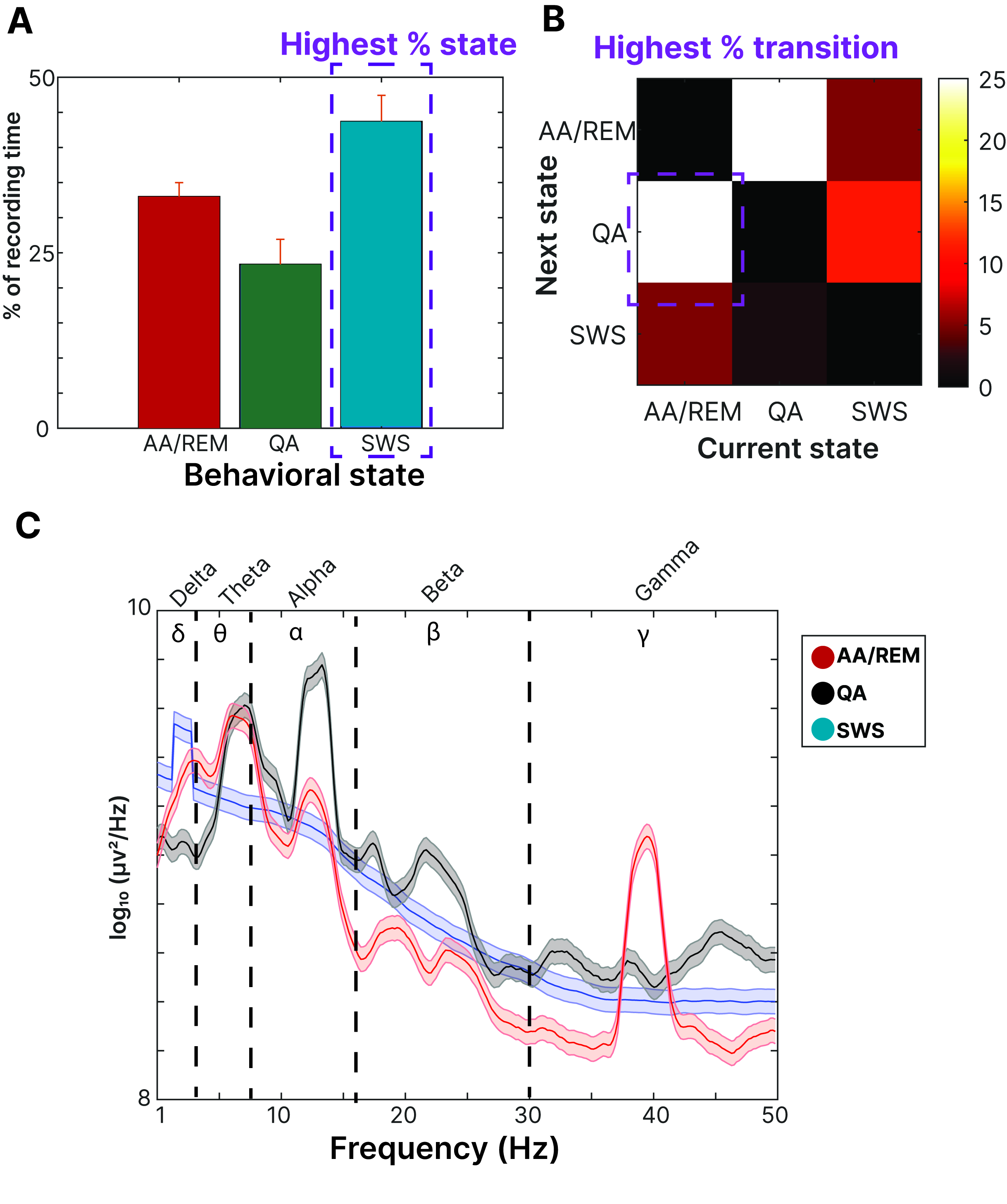

Supplement: S1 Fig — Description of relative recording time, transition between states and predominant frequencies: (A) The percentage of the recording time indicates the SWS as the behavioral state with the highest percentage while (B) the transition probability matrix indicates the transition from AA/REM to QA as the one that occurred the most amount of times. (C) The power spectrum displayed the predominant frequencies for each of the behavioral states. (TIF) [file pcbi.1011818.s001.tif]
