# Supplementary material for: Functional hierarchies in brain dynamics characterized by signal reversibility in ferret cortex
Source: PLoS Comput Biol. 2024 Jan 19;20(1):e1011818. doi: 10.1371/journal.pcbi.1011818 (PMC10836715; doi:10.1371/journal.pcbi.1011818)
Supplement: Attachment — Submitted filename: Reviewer_Report_Plos.pdf [file pcbi.1011818.s003.pdf]

Referee Report, manuscript PCOMPBIOL-D-23-01038.

November 30, 2023

Title : *Functional hierarchies in brain dynamics characterized by signal reversibility in ferret cortex*

Submitted for publication in the Journal PLOS Computational Biology

1 Manuscript overview

This manuscript explores brain signal irreversibility, which is as a promising avenue for understanding neural dynamics. It studies brain signal irreversibility in relation to cortical hierarchy and the influence of various electrophysiological features. Local field potentials (LFPs) were recorded in ferrets during spontaneous behavior using custom micro-electrocorticographic arrays. Diverse metrics were employed to measure complexity levels in different behavioral states, with a focus on brain irreversibility to quantify non-equilibrium captured by the arrow of time in the signal.

The findings reveal a hierarchical organization of the ferret's cortex through different signatures of irreversibility in three brain states (active awake, quiet awake, and deep sleep). Deep sleep in ferrets exhibits low entropy production compared to awake states, a result in agreement with recent results in macaques (Sanz-Perl 2021), and humans (Gilson 2023). The study further disentangles the influence of different brain regions and frequency bands, highlighting the predominance of the parietal area and the theta band in the irreversibility process. The application of a Hidden Markov Model exposes lower switching rates and entropy production during the deep sleep stage, suggesting functional hierarchies in organizations that can be unveiled through thermodynamic features and information theory metrics.

In my assessment, this work is interesting, but not well-articulated. The current form presents several mistakes that show a partial understanding of the concept of entropy production and time-reversal asymmetry, and its relationship with thermodynamics. In my view, the manuscript should address both major and minor issues before a more serious consideration for publication in PLOS Computational Biology.

2 Major

- In the introduction you mention *"One promising analysis framework considers the description of brain states in terms of brain dynamics operating out of equilibrium, based on time series reversibility features (Deco et al., 2022; Lynn et al., 2021)."* The list is much bigger and interesting, you should mention the recent articles of de la Fuente et al., 2023, Sanz-Perl et al., 2021, Gilson 2023, Cruzat 2022, etc..

- It is not clear how the irreversibility level was calculated. You mention overlapping sliding windows (of 1 second), but you do not mention the overlap. Both quantities must be specified.

- It is not clear what the time series $x(t)$ and $y(t)$ are in your example. Two signals from two electrodes?

- When you mention detailed balance, you seem to not clearly state what this condition means. For example, for a Markov chain, it is not that the probability of observing a transition between any given two states in the configuration space is the same as the probability of observing the reverse transition. The detailed balance condition is a series of equations that

require that that the transition probability matrix P for the Markov process that possess a stationary distribution π satisfies that

$$\pi_i P_{ij} = \pi_j P_{ji}$$

for all i, j.

- Your equation 4 is wrong if you consider forward and backward transition probabilities. It is only correct if you consider joint probabilities of two consecutive time steps, i.e., $P_{xy} = \pi_x P_{x \to y}$
- You mention "if the system follows detailed balance" I would say satisfies the detailed balance conditions...the transition probabilities between any two states are equal to the reverse probabilities...again this is wrong... and then the entropy production will be zero.. for this you have to assume that $0 \log 0 = 0$, which is not explicitly stated in the equations.
- In the section results, you mention "We inspected the temporal asymmetry of the dynamical system", which dynamical system? seems to me that you only look at the experimental time series without referring to any dynamical system.
- Then you mention that you measure non-reversibility and consequently the level of non-equilibrium of the system. While for some non-equilibrium steady states found in statistical mechanics, this relationship holds, it is not clear to me what you mean here. What is the equilibrium state from which you measure the level of non-equilibrium of the system?
- In the results you mention "If the relation between the network states was the same in both directions (symmetrical), then the entropy level would be 0." Again you are confusing entropy with entropy production.
- Fig 3f, you show transition probabilities but the values that you show reveal that these are not transition probabilities, as the sum of each row is not one.
- In the discussion you mention "We show how this measure of irreversibility can be used as a proxy of non-equilibrium", Actually you don't show that, you only vaguely mention it.
- In the discussion you mention "Previous studies have inspected non-equilibrium of signals in macaque monkeys (Deco et al. 2022)" You forgot one relevant article by one of the authors of this manuscript (Sanz Perl 2021).
- In the discussion you mention that the SWS behavioral state exhibited a lower entropy production, with respect to the awake state. This result is in agreement with the recent result obtained using human fMRI data (Gilson 2023), a relevant relationship may appear here, you should compare and comment on this.
- In the discussion you mention " In conclusion, we demonstrated how to direct metrics derived from thermodynamics such as signal reversibility". Your custom measure of signal reversibility (equation 3) seems not to be derived from thermodynamics.

3 Minor

- In the definition of determinism you mention "The average determinism of a directed network X with N .. with N what?
- In the definition of degeneracy, it is not clear to me how you compute the entropy of an average. Is the distribution of averages? Also, seems that a parenthesis is not necessary and you define a network as X and then refer to it as x.
- In the definition of mutual information, the definition itself is missing, you only mention properties and mention "strictly independent" while being independent is a property that a pair or random variables holds or not. Statistical interdependence is different.

- In the results you mention "The absolute difference between the irreversibility matrices (forward minus backward)" what do you mean by irreversibility matrices, seems to me that you compute the difference between the functional connectivity matrices.
- Fig 1c what is the time scale? After you mention that you compute irreversibility values (difference between forward and backward matrix) at each time point. It is not clear at all how this quantity is computed following your definition in Equation 3. Is an average over all pairs?
- How do you explain the differences between irreversibility and entropy production between panels C and I?
- In the discussion you mention "the extensive recording time (>3 hours per animal)". This should appear before not in the conclusion. Before you only mentioned resting activity during the light cycle between 12 a.m. and 6 p.m., but is not clear if you used all the data.
- If you want to illustrate a transition probability a color bar with the values is missing.
- In figure 1 is not clear what HMM-MAR brings to the irreversibility framework.
- In the caption of panel I) you mention "The highest entropy" do you mean entropy production?
- Figure 2B is badly explained. Took me a while to understand what you did and I'm not sure I understood. You should be more explicit on what each column means. Only the color of the rectangle guides what you mean. Also, you should be more explicit in the x-axis of the histograms. It is not clear how you measure irreversibility between and within regions
- In my opinion, the computational code used to obtain the results should always be available, readable, and usable without even needing to request it.
