# Supplementary material for: Functional hierarchies in brain dynamics characterized by signal reversibility in ferret cortex
Source: PLoS Comput Biol. 2024 Jan 19;20(1):e1011818. doi: 10.1371/journal.pcbi.1011818 (PMC10836715; doi:10.1371/journal.pcbi.1011818)
Supplement: Attachment — Submitted filename: Response_to_reviewers_Idesis_Functional_Hierarchies.docx [file pcbi.1011818.s002.docx]

Dear Editor and Reviewers:

We thank you for the helpful feedback we have received on our manuscript. This letter contains our responses to each of the reviewers’ comments. Our responses are provided below as indented paragraphs in blue, quotes or additions to the manuscript text are in green, and portions of the original text are in light green.

On behalf of all co-authors,

Sebastian Idesis, MSc.

We benefited from the feedback provided by the editor and the reviewers which helped to improve the clarity and completeness of the manuscript. Among the changes made, we have incorporated missing statistics reports (both graphically and numerically), controlled for potential confounds in different analysis, explained the methodology in a more detailed way and expanded the discussion for a higher clarity. We hope the outcome of this revision is a more understandable and detailed presentation of the performed study. In the next pages, we answer each of the questions posed by the reviewers:

***Reviewer 1****: In "Functional hierarchies in brain dynamics characterized by signal reversibility in ferret cortex", Idesis et al. describe an analysis of μECoG data in ferrets that was described previously in Stitt et al. (2017). In the current analysis, the authors compare temporal irreversibility across different behavioral states (i.e., active awake, quiet awake, asleep), and further relate it to different metrics of brain activity dynamics.*

*The authors provide interesting results on how temporal irreversibility, a proxy for broken detailed balance, is related to transient temporal dynamics (HMM) and brain region. My biggest concerns are in regards to missing methodological details, motivations for using specific parameters, methods, and comparisons, and interpretation. Please see my comments below.*

We thank the reviewer for this kind feedback and for taking the time to consider our manuscript. We appreciate the encouraging comments and insight.

***Major:***

*1. a) In the HMM pipeline, the authors take the first principal component of each brain region. Firstly, they do not provide details on the different brain regions, nor how many electrodes are in each region – on a closer inspection, it appears this information is present in Figure 3. I suggest the authors restructure this figure such that this information can be found earlier in the paper.*

We thank the reviewer for commenting on this topic. We have modified Figure 1a to include the missing information and adapted the corresponding legend in order to clarify the presented figure.

Figure 1: Analysis summary: (A) Data was recorded from 64 custom micro-electrocorticographic (μECoG) electrodes placed in the ferret cortex. The number of electrodes in each cortical functional system (auditory, visual, parietal) is depicted in the insert, which sum to 64 total electrodes.

*b) Secondly, after the PCA step, it is unclear whether/how the authors deal with spatial leakage (zero-lag correlations) before fitting the HMM.*

We appreciate the comment of the reviewer. We have added a sentence explaining how this potential confound was taken into consideration during the analysis in the HMM methods section. We note that this correction method has been previously validated and applied by the creators of the HMM toolbox and have cited their relevant publications:

In order to attenuate the effect of possible spatial leakage (due to the adjacency of neighboring areas), symmetric multivariate leakage correction was applied across the whole network as proposed by the creators of the used toolbox (Colclough et al., 2015; Quinn et al., 2018).

*c) the authors do not explain or motivate why they chose to look at the two summary metrics of the HMM: switching rate, and maximal fractional occupancy. Especially the choice of the latter is unclear to me, as is its definition.*

We thank the reviewer for pointing out this issue. We have added a sentence justifying the election of the metrics

We selected these metrics as they have been reported extensively in previous literature and suggested as metrics of analytical interest by the toolbox used (Baker et al., 2014; Quinn et al., 2018; Vidaurre et al., 2016).

Furthermore, we expanded the definition of “Maximal fractional occupancy” in the corresponding methods section

In other words, fractional occupancy is defined as the fraction of time spent in each state:

$$Fractional occupancy \left( k \right)= \frac{1}{T} \sum_{t} (u_{t}==k)$$

(5)

Where, in the state space dimension K, the most probable a posteriori state ($u_{t})$ is one if $u_{t}=k$ and is zero otherwise, and T is the length of the network state sequence in samples (Baker et al., 2014).

*d) are the differences in the HMM metrics between behavioral states significant?*

We appreciate the comment from the reviewer and agree that the figure could be more explanatory. Therefore, we modified figures 1e and 1f in order to include the corresponding significance.

Furthermore, we added a report of the observed statistics in these metrics in the results section:

There was a significant difference between the three behavioral states ([F(2, 9)=64.18,p<.01]) where AA/REM had the highest level of switching rate (0.155), followed by QA (0.133) and SWS (0.124) (Figure 1E). The reversed pattern was observed when looking into the maximal fractional occupancy (“MaxFO”, the highest value of time staying at each network state). There was a significant difference between the three behavioral states ([F(2, 9)=23.34,p<.01]) where SWS displayed the highest MaxFO (0.44), followed by QA (0.37) and then (0.32) (Figure 1F).

*e) while the authors comment on having provided insights into how irreversibility relates to HMM dynamics, it is unclear what the meaning of these commonalities is, which I believe is restricted to the same or reverse pattern of irreversibility, switching rate, and max FO for the three behavioral states.*

We thank the reviewer for pointing out this doubt. We have expanded the discussion section in order to clarify this issue.

The obtained results demonstrate how a common underlying mechanism which is altered in different behavioral states as reported in previous literature (G-Guzmán et al., 2023) can be captured in diverse ways, including direct metrics, such as irreversibility or indirect calculations through dimensionality reduction, such as HMM or the use of autoencoders.

*2. a. From text in the results (P14L5) it is unclear what the authors wish to achieve with the use of the deep autoencoder, and how the entropy production at the latent dimension of 7 is related to the irreversibility. Especially considering the HMM results, where the authors show congruent effects of irreversibility and summary metrics on only 5 states (i.e., an even lower dimension than 7).*

We thank the reviewer for the question. We have expanded the discussion section in order to clarify this issue.

By presenting results obtained through HMM and autoencoder dimensionality reduction, we show convergent evidence that strengthens the hypothesis framed in this article. The alternative approaches show different features of the same underlying phenomena, providing robust techniques for its exploration. Furthermore, it shows the association between entropy production and time-irreversibility, as it has been previously proposed (Qian et al., 2002; Sagawa, 2019).

*b. Furthermore, they claim that “the emerging distributions match the pattern observed in the network states of the HMM and in the irreversibility at the source space”, while this only is the case for AA/REM having the highest value – QA and SWS show the reverse pattern.*

We thank the reviewer for bringing up this issue. We have modified our statement to avoid confusion.

When computing the total entropy production in this dimensionality, the AA/REM showed the highest level of entropy, as observed previously in the switching rate of network-states of the HMM and in the irreversibility analysis at the source space (Figure 1I).

*3. The authors investigate the within and between region irreversibility for each region. However, neighboring electrodes are likely to have a higher correlation due to spatial leakage, and this could be affected by the configuration and number of electrodes in each region. Could the authors comment on the effect of this cross-correlation on irreversibility, and the potential confound this could be for investigating between region differences?*

We agree with the reviewer on the possibility of expanding the connection between these concepts. Therefore, we incorporated to the methods and results section, a clarification about these topics:

In order to attenuate the effect of possible spatial leakage (due to the adjacency of neighboring areas), symmetric multivariate leakage correction was applied across the whole network as proposed by the creators of the used toolbox (Colclough et al., 2015; Quinn et al., 2018).

The observed pattern was consistent disregarding the different number of electrodes per system (Figure 1a). The level of irreversibility was higher in the within-system relations in all the states (AA/REM: .0069; QA: .0057; SWS: .0074) compared to the between-system relations (AA/REM: .0045; QA: .0052; SWS: .0063). It is important to clarify that existing literature has already proven how the cross-correlation of neighboring cortical areas is a genuine feature of the data and does not reflect statistical noise. In this way, the cross-correlation and spatial leakage (see methods) would not be potential driving factor for the estimation of the signal irreversibility (Tewarie et al., 2023).

*4. In the section “Embedded network system revealed different entropy dynamics according to the behavioral state”, the authors return to the comparison of irreversibility and the HMM that they started in the first section of the Results. This order of the results is hard to follow, and hampers readability (as mentioned in point 1). I recommend the authors to consider reorganizing the results.*

We thank the reviewer for the suggestion. We have re-written the first paragraph of the mentioned section in order to improve readability.

The behavioral states dynamics can be inspected through the observation of the transition between them. We revisit the results obtained in Figure 1d to extend the analysis. In this section we aim to reconnect the results presented previously with several metrics obtained through the observation of the transitions across the different network states.

Using the HMM framework, we obtained 5 network substates and inspected information theoretical metrics in order to study if the dynamics between the substates are consistent with the previously reported results.

*5. In figure 3E-I, the authors compare the entropy production (irreversibility of HMM states) with three other information theoretic metrics, and show that these are consistent. However, it is unclear how entropy production provides more insights into the neural dynamics, and generally, what the significance is of differences in irreversibility throughout the paper.*

We thank the reviewer for this comment. We have extended the corresponding result section in order to enhance the clarity of this result interpretation:

By incorporating these metrics, we show converging evidence that this phenomenon is supported by several lines of evidence. Through coherent results obtained in the distinct metrics, the underlying brain dynamics can be explored in order to shed light on this topic.

***Minor:***

*1. The authors are currently plotting differences between behavioral state in bar plots. While these are easy to read and can clearly show differences in mean between conditions, they do not display the underlying distributions of the data (see https://doi.org/10.1371/journal.pbio.1002128). Therefore, I recommend the authors use box or violin plots instead.*

We thank the reviewer for the suggestion. We have taken into consideration the proposal and modified the figure 1c in order to display the underlying distribution of the data (We used boxplot to keep the consistency across the remaining figures):

*2. In P11L11, the authors write “Posteriorly, all 64 spectrograms were averaged into a single global spectrogram.” But later in the same section “By means of PCA the dimensionality was reduced to the 8 strongest components…”. Please clarify that (presumably) the PCA was run on the entire 64 channel data.*

We appreciate the suggestion of the reviewer. We have modified the mentioned sentence in order to enhance readability

By means of PCA (applied on the entire 64 channel data), the dimensionality was reduced to the 8 strongest components on which a k-means cluster analysis was applied to classify each recording session into 3 clusters.

*3. In the Methods, the definition of “< ( ) >” and “ < ( ) >” are written very concisely, but could further explained.*

We thank the reviewer for the comment. We have expanded the definition of the average used across the manuscript

Furthermore, the arithmetic mean (represented in the formulas as <> can be calculated by

Arithmetic Mean = $\frac{\sum_{i}^{n} x_{i}}{n}$

(3)

*4. P13L24: The authors write “The frequency spectrum was used to determine the behavioral state across the whole recording”. Please add a plot of the spectrum of the three behavioral state (as a supplement), i.e., similar to Figure 3B in Stitt et al.*

We thank the reviewer for the suggestion. We have added in the supplementary figures a similar figure as the one proposed while also including the legend of the supplementary figure added.

(C) The power spectrum displayed the predominant frequencies for each of the behavioral states.

*5. P13L21: It is unclear how the authors compute the irreversibility metric (fig. 1C) from the sensor x sensor irreversibility matrix (Fig 1B), i.e. do they average the irreversibility over channels ?*

We are grateful with the reviewer for pointing this potential misunderstanding. We have added a sentence in the result section to enhance clarity:

In other words, the irreversibility metric was calculated over all channels (by assessing the difference in the corresponding matrices, see methods), providing one unique reversibility value for each time point.

*6. Figure 1D: it is unclear what the different colors correspond to. Please provide a color bar.*

We thank the reviewer for the comment. We modified the figure to avoid confusion.

***Reviewer 2****: Thank you for inviting me to review this very interesting manuscript by Idesis and colleagues, using several markers of dynamics from thermodynamics and information theory to study ferret micro-ECoG traces across multiple behavioural states. This is a very interesting study, combining high-resolution data from a rarely-studied species, with a battery of interesting ways of characterising brain activity. The topic is of interest and timely. However, I have some reservations about the statistical reporting, which seems to be nearly absent. Discussion is instead primarily based on numerical differences. Even when statistical results are reported as non-significant, this non-significance is then simply ignored, instead repeatedly claiming that the results are consistent with previous observations when in fact the null hypothesis was not rejected. Other claims are at present difficult to evaluate because statistics are simply not provided, as far as I can determine. So my main recommendations are to add a section describing the statistical approach in detail; provide the statistics themselves in the text, and error bars in the figures; and then respect the meaning of the statistical results in the discussion and interpretation, without interpreting merely numerical differences. I believe that doing so can make for a compelling manuscript, even if the results were to be negative.*

We thank the reviewer for highlighting this issue. We added the corresponding statistical report in detail. We also stress the distribution and error bars in the corresponding plots in order to increase the interpretation of them. In the next pages, we answer each of the questions posed by the reviewer:

*1.* *Page 3: “Furthermore, the obtained results can be compared and validated with embedded dynamics that can be obtained by dimensionality reduction approaches and generative models of switching behavior.” It would be helpful to explain why these approaches are desirable, in addition to merely stated that they can be applied.*

We thank the reviewer for this comment. We have extended the interpretation in order to include the usefulness of the proposed metrics.

Furthermore, the obtained results can be compared and validated with embedded dynamics that can be obtained by dimensionality reduction approaches and generative models of switching behavior. In this way, we propose an easy and fast to implement approach, comparable to already validated techniques which reduce the noise in the signal and remove the redundancy of high-dimensional neuroimaging recordings.

*2. “these data pose a unique analytical challenge – combining high 1 temporal resolution neural signals with tracking of much slower behavioral changes. Can the extracted time series metric, such as irreversibility, that is applied to the finely sampled neural recordings, reveal a precise transition between changing behaviors?”*

We thank the reviewer for this comment. We have assessed the irreversibility levels at each time point providing evidence on how the transition between states is described, instead of just comparing the average in the different groups (Figure 2a). Furthermore, previous literature have already tackled this issue showing evidence of the benefits of use high temporal resolution recordings for this type of analysis (Masaracchia et al., 2023; Tewarie et al., 2023)

*3. Did the authors not already apply irreversibility to ECoG sleep data of the macaque? It would be helpful to clarify how the present study differs from that study (since both involve sleep data from non-human animals sampled at high temporal resolution). Otherwise the question appears a bit of a straw-man. The new species is a clear asset to the paper, so this may well be mentioned.*

We thank the reviewer for this pointing this topic. We have expanded this section in order to provide more evidence of the strengths of this study in compared to the one reported in macaques.

Previous studies have inspected non-equilibrium of signals in macaque monkeys (Deco et al., 2022), allowing to disentangle different consciousness levels through the non-reversibility of the signal. Nevertheless, the distinction between the brain states was due to pharmacological intervention instead of occurring spontaneously as in the present paper. Our study benefits from the relatively short duration of behavioral states in the ferret model and the extensive recording time (>3 hours per animal) in order to study the transition between the different states. Besides the significant difference in the recording durations, our study consists of continuous recordings from the same animals, allowing the exploration of the transition between the states, in contrast to previous studies, where the recordings of the different states were discrete and recorded in different periods of time.

*4.* *It is not clear what are the variables between which mutual information is being computed. Could the authors clarify this in the Methods?*

We appreciate the comments of reviewer. We added a paragraph in the corresponding method section to clarify the use of this metric.

The quantification of mutual information exposes information between the future and past joint states of the variables that make up a network. Therefore, the calculation of mutual information quantifies how much knowledge of the past state of the system reduces our uncertainty about the future state of the system (Varley & Hoel, 2022).

*5.* *“([F(2, 3660893)=83627,p<.01]” is the significance merely a result of an extremely high degrees of freedom? In fact, what test is this? The statistical approach does not seem to be described anywhere in the paper. It would be inappropriate to treat each instance of 1s-long recording as an independent observation, since they are in fact provided by the same animals. The statistical approach should account for this non-independence in the data, bot across conditions/physiological stages but also within each condition (same animal providing multiple observations).*

We thank the reviewer for the comment. We improved our paragraph by making explicit the statistical analysis performed and the further correction.

Using a repeated-measures ANOVA (as the samples are from the same recording), we found that there was a significant difference in the irreversibility level between all the behavioral states ([F(2, 3660893)=83627,p<.01] being “Active-awake/REM (AA/REM)” sleep the state with the highest value of irreversibility (mean=0.05 +/- 0.002), followed by “Quiet-awake (QA)” (mean=0.03 +/- 0.002) and “Slow wave sleep (SWS)” (mean=0.02 +/- 0.001) (Post-hoc Bonferroni corrected) (Figure 1C).

Regarding the possibility of this phenomenon being explained by the high degrees of freedom, we provide a supplementary analysis in which the same analysis was applied to hundreds of randomly extracted subsamples (25 presented in the figure for visualization purposes) of lower temporal dimension (1000 time points each). The result shows a significant difference between the three behavioral states in all the subsample comparisons, probing the low influence of the degrees of freedom.

*6. “The level of irreversibility was higher in the within-region areas in all the states 29 (AA/REM: .0069; QA: .0057; SWS: .0074) compared to the between-region areas (AA/REM: 30 .0045; QA: .0052; SWS: .0063).” Is this only a numerical difference, or is it statistically reliable? I could not find statistical reporting, and Figure 2 also seems to lack error bars.*

We thank the reviewer for pointing out this topic. We have updated figure 2b to include the corresponding distribution and significance references. We have also added the statistical report into the text.

The observed pattern was consistent disregarding the different amount of electrodes per system (Figure 1a). By means of paired t-student comparisons, we found a significant difference between the within-system and between-system irreversibility values (p<.01). The level of irreversibility was higher in the within-system relations areas in all the states (AA/REM: .0069; QA: .0057; SWS: .0074) compared to the between-system relations (AA/REM: .0045; QA: .0052; SWS: .0063).

*7.* *Page 20, ln. 26-30: are the associated statistics for these results provided anywhere? As far as I can tell, only numerical differences are reported, and the figure suggests that at least some of the measures are not statistically different between conditions. So as it stands, no inferences can be made based on these measures.*

We apologize to the reviewer for this omission. We clarified in the results section the lack of significance in the comparison between the states, explained mostly but the low sample size (usual in this kind of study). Furthermore, we updated the figures to include the lack of significant differences

Despite showing a consistent trend, in all the information theory metrics (Figure 3G-H-I), the difference between the states was not significant (p >.13) mostly due to the low number of animals considered for the analysis.

*8. Page 20, “AA/REM showed the highest entropy production”. This is numerically true but statistically indistinguishable from random chance, so it is not appropriate to describe it as consistent with the irreversibility analysis: the results are in fact unable to reject the null hypothesis that behavioral state affects entropy production.*

We thank the reviewer for the comment. We have modified figure 1E to include the significance of the comparison while also correcting the text to incorporate the missing statistical report. Reporting the significant different difference between two of the states (while the other two comparisons were not significant). As the result shows a significant difference with the null hypothesis, it justified its use in further discussion.

There was a significant difference between the different states ([F(2, 9)=4.79,p<.05]) being the difference between AA/REM and SWS, the highest one (p=.03). The AA/REM had the highest entropy production level (mean=0.0135, std=0.0079), followed by QA (mean=0.0118, std=0.0075) and lastly SWS (mean=0.0087, std=0.0054) (Figure 3E).

*9.* *“observed a congruent pattern with previous literature comparing different levels of complexity according to the corresponding brain state (Varley et al., 2021).” I apologize if I missed this, but as far as I can determine the only measure for which any statistics are reported is the entropy production, which however was not significantly different across conditions. So in fact it is not appropriate to treat it as being consistent with the irreversibility results or with prior literature, since the difference is not distinguishable from chance, and the null hypothesis that state of wakefulness has an effect on entropy production is not rejected.*

We thank the reviewer for the comment. We have corrected the report of entropy production showing its significant difference. Furthermore, we have added the statistical report of the missing metrics. Lastly, we have corrected the reported statement to avoid confusions:

We applied several information-theoretic metrics to the network states obtained through the HMM analysis and observed a congruent trend (despite not being significant due to the low sample size) with previous literature comparing different levels of complexity according to the corresponding brain state (Varley et al., 2021).

*10. The same concern applies to lines 16-18 of page 26, where it is claimed that “we demonstrated how direct metrics derived from thermodynamics such as signal reversibility reflect brain dynamics also captured by statistical models and information theory metrics.”. The last part of this sentence is something that the authors in fact specifically failed to demonstrate, so this statement is misleading. Even if a significant difference is not found, the irreversibility results hold and are of interest in themselves.*

We thank the reviewer for the comment. We have improved the mentioned paragraph to make for clear the conclusions and avoid potential misunderstandings for the readers.

In conclusion, we demonstrated how direct metrics derived from thermodynamics such as signal reversibility reflect brain dynamics distinguishable between different behavioral states. Furthermore, we found similar results (coherent trends following the same pattern) captured by statistical models and information theory metrics.

Baker, A. P., Brookes, M. J., Rezek, I. A., Smith, S. M., Behrens, T., Probert Smith, P. J., & Woolrich, M. (2014). Fast transient networks in spontaneous human brain activity. *Elife*, *3*, e01867.

Deco, G., Sanz Perl, Y., Bocaccio, H., Tagliazucchi, E., & Kringelbach, M. L. (2022). The INSIDEOUT framework provides precise signatures of the balance of intrinsic and extrinsic dynamics in brain states. *Communications Biology*, *5*(1), 1-13.

Masaracchia, L., Fredes, F., Woolrich, M. W., & Vidaurre, D. (2023). Dissecting unsupervised learning through hidden Markov modelling in electrophysiological data. *BioRxiv*, 2023.2001. 2019.524547.

Qian, H., Qian, M., & Tang, X. (2002). Thermodynamics of the general diffusion process: time-reversibility and entropy production. *Journal of statistical physics*, *107*, 1129-1141.

Quinn, A. J., Vidaurre, D., Abeysuriya, R., Becker, R., Nobre, A. C., & Woolrich, M. W. (2018). Task-evoked dynamic network analysis through hidden markov modeling. *Frontiers in neuroscience*, *12*, 603.

Sagawa, T. (2019). Second law, entropy production, and reversibility in thermodynamics of information. *Energy Limits in Computation: A Review of Landauer’s Principle, Theory and Experiments*, 101-139.

Tewarie, P. K., Hindriks, R., Lai, Y. M., Sotiropoulos, S. N., Kringelbach, M., & Deco, G. (2023). Non-reversibility outperforms functional connectivity in characterisation of brain states in MEG data. *Neuroimage*, 120186.

Varley, T. F., Denny, V., Sporns, O., & Patania, A. (2021). Topological analysis of differential effects of ketamine and propofol anaesthesia on brain dynamics. *Royal Society open science*, *8*(6), 201971.

Vidaurre, D., Quinn, A. J., Baker, A. P., Dupret, D., Tejero-Cantero, A., & Woolrich, M. W. (2016). Spectrally resolved fast transient brain states in electrophysiological data. *Neuroimage*, *126*, 81-95.
