# Supplementary material for: Functional hierarchies in brain dynamics characterized by signal reversibility in ferret cortex
Source: PLoS Comput Biol. 2024 Jan 19;20(1):e1011818. doi: 10.1371/journal.pcbi.1011818 (PMC10836715; doi:10.1371/journal.pcbi.1011818)
Supplement: Attachment — Submitted filename: Response_to_reviewers_Idesis_Functional_Hierarchies.docx [file pcbi.1011818.s004.docx]

Dear Editor and Reviewers:

We thank you for the helpful feedback we have received on our manuscript. This letter contains our responses to each of the reviewers’ comments. Our responses are provided below as indented paragraphs in blue, quotes or additions to the manuscript text are in green, and portions of the original text are in light green.

On behalf of all co-authors,

Sebastian Idesis, PhD.

We benefited from the feedback provided by the editor and the reviewers which helped to improve the clarity and completeness of the manuscript. Among the changes made, we have incorporated missing statistics reports, checked for potential confounds in different analysis, explained the methodology in a more detailed way and expanded the discussion for higher clarity. We hope the outcome of this revision is a more understandable and detailed presentation of the performed study. In the next pages, we answer each of the questions posed by the reviewers:

***Reviewer 2:***

1) I wish to thank the authors for taking my feedback into account, and for their additional work. However, I still have concerns about the appropriateness of the statistical testing and reporting. I tried to access the data from the link provided by the authors, to have first-hand understanding of the data organization, but it seems that only a single 64x1200 matrix is shared. I would also encourage the authors to add a dedicated Statistics section in the Methods that explains the statistical approaches in detail. I outline my detailed feedback on the revisions below.

We thank the reviewer for bringing up this topic. We had uploaded the aforementioned 64x1200 fake matrix with the purpose of running and testing our provided scripts. Regarding the real animal data, we will make the recordings used in our analysis available after acceptance of the paper. The data will be made available in the research data repository of the University of Hamburg (https://www.fdr.uni-hamburg.de). This will be with restricted access, i.e., access to other researchers is granted after providing identity of the person requesting the download and information on the purpose of the intended use

Furthermore, we have added a statistics section in the methods in order to enhance clarity.

Statistics

We performed paired statistical analyses (t-test/ANOVA, depending on the group number) as the comparison was performed for the same animal in different brain states. Animal ID was added as a random effect to control the violation of independence of the data. Statistical significance was assessed with a threshold of p=.05. All post hoc comparisons were Bonferroni corrected for the number of comparisons taking place. The code for obtaining the metrics and estimation of the results can be accessed in the following link.

Code and sample data are available at –

<https://github.com/SebastianIdesis/Hierarchy_Ferrets-2023->

2) Response to Comment #4:

I thank the authors for the clarification of what mutual information is. However, the added text still does not explain what exactly is used as each variable, in the present case.

We thank the reviewer for the comment. We have expanded the explanation of the concept to make clearer the used variables.

Therefore, the calculation of mutual information quantifies how much knowledge of the past state of the system reduces our uncertainty about the future state of the system (Varley & Hoel, 2022). As a result, mutual information is calculated by adding the information (entropy) of the previous and future state and subtracting the joint entropy of them.

3) Response to Comment #5:

I am not convinced that a repeated-measure ANOVA is the appropriate test here. The ANOVA will (correctly) take into account that the same animals provide data for multiple conditions (AA/ Rem, SWS), but it will not take into account the fact that the same animals also provide data *within* each condition: this is an additional source of non-independence in the data that needs to be accounted for. The same issue would also apply to the new analysis with random subsamples of 1000 timepoints: the 1000 timepoints are not independent because there were not 1000 animals, so some timepoints will come from the same animal, violating the assumption of non-independence. Adding animal ID as a random effect would be one way of controlling for this violation of independence. Another way would be to average all data that come from the same animal, within each condition.

We thank the reviewer for the suggestion. We have modified our analysis and replaced it with a mixed effect ANOVA including the animal ID as a random effect. We have updated the corresponding statistical reports

4) Response to Comment #6:

The figure is very helpful, thank you. However, since box-plots are used, it is not clear how many data-points were used. Throughout the text, the authors should report the statistics in full (t-score, degrees of freedom, and effect size), not just the means and p-value.

We thank the reviewer for the suggestion. We have added the report of the statistics as requested by the reviewer:

By means of paired t-student comparisons, we found a significant difference between the within-regions (Connections between brain areas of the same regions) and between-regions (Connections between brain areas of the different regions) irreversibility values (p<.01). The difference was significant at AA/REM state (t(4095)= 215.30, p< .01), QA (t(4095)= 215.73, p< .01) and SWS (t(4287)= 225.86, p< .01).

Furthermore, we have added the corresponding statistical reports where appropiate.

5) Response to Comment #7:

Bayesian statistics could be used to tell whether the difference is due to insufficient power (as the authors reasonably imply) or whether there is actual support for the null hypothesis.

We thank the reviewer for the suggestion. We agree that an extra analysis is needed to investigate the certainty for supporting the null hypothesis. Therefore, we performed the Bayesian statistics, showing the certainty at each of the mentioned metrics. Through 100 iterations of the analysis, we have reported the percentage of cases in which the null hypothesis was confirmed. As our claim is inferential, we declared no clear evidence of the phenomena while stating that the inclusion of more animals would allow the confirmation of this hypothesis. Scripts used for this analysis are provided in the GitHub page mentioned in the manuscript.

An extra analysis, through a Bayesian ANOVA (https://github.com/klabhub/bayesFactor) showed a high level of certainty in the three metrics confirming the support for the null hypothesis (Certainty: Determinism 84%, Degeneracy 90%, Mutual information: 84%).

6) Response to Comment #7:

Is the significance adjusted for all the multiple comparisons (pairwise t-tests) performed? This won’t affect the presence of overall significance from the ANOVA, but would still be appropriate when interpreting the pairwise differences. As before, I would encourage the authors to show the statistical results in full in the text or figure caption (t-score, degrees of freedom, effect size), not just the p-value, and not just for significant results.

We thank the reviewer for the comment. We have added a clarification for the multiple comparisons and the corresponding statistical results.

There were no significant differences neither in determinism ([F(1, 10)=0.06,p=.79]), nor degeneracy ([F(1, 10)=1.08,p=.32]), nor mutual information ([F(1, 10)=0.01,p=.89) (multiple comparisons were not significant and addressed via Bonferroni correction).Response to Comment #9:

7) As before, Bayesian statistics could be used to tell whether the difference is due to insufficient power or whether there is actual support for the null hypothesis.

We thank the reviewer for the suggestion. As commented in a previous answer, we have added the Bayesian statistics of the discussed metrics.

***Minor***

8) Figure in response to Comment #6: there is the word “path” in violet, which the authors may wish to remove.

We thank the reviewer for pointing out this topic. We have corrected the figure in the manuscript removing the word “path”

***Reviewer 3****:*

This manuscript explores brain signal irreversibility, which is as a promising avenue for understanding neural dynamics. It studies brain signal irreversibility in relation to cortical hierarchy and the influence of various electrophysiological features. Local field potentials (LFPs) were recorded in ferrets during spontaneous behavior using custom micro-electrocorticographic arrays. Diverse metrics were employed to measure complexity levels in different behavioral states, with a focus on brain irreversibility to quantify non-equilibrium captured by the arrow of time in the signal.

The findings reveal a hierarchical organization of the ferret’s cortex through different signatures

of irreversibility in three brain states (active awake, quiet awake, and deep sleep). Deep sleep in

ferrets exhibits low entropy production compared to awake states, a result in agreement with recent

results in macaques (Sanz-Perl 2021), and humans (Gilson 2023). The study further disentangles

the influence of different brain regions and frequency bands, highlighting the predominance of

the parietal area and the theta band in the irreversibility process. The application of a Hidden

Markov Model exposes lower switching rates and entropy production during the deep sleep stage,

suggesting functional hierarchies in organizations that can be unveiled through thermodynamic

features and information theory metrics.

In my assessment, this work is interesting, but not well-articulated. The current form presents

several mistakes that show a partial understanding of the concept of entropy production and timereversal asymmetry, and its relationship with thermodynamics. In my view, the manuscript should address both major and minor issues before a more serious consideration for publication in PLOS Computational Biology.

We thank the reviewer for this kind feedback and for taking the time to consider our manuscript. We appreciate the encouraging comments and insight.

***Major:***

1) In the introduction you mention "One promising analysis framework considers the description of brain states in terms of brain dynamics operating out of equilibrium, based on time series reversibility features (Deco et al., 2022; Lynn et al., 2021)." The list is much bigger and interesting, you should mention the recent articles of de la Fuente et al., 2023, Sanz-Perl et al., 2021, Gilson 2023, Cruzat 2022, etc..

We thank the reviewer for the suggestions. We have updated the references to include the suggested articles

features (Cruzat et al., 2023; de la Fuente et al., 2023; Deco et al., 2022; Gilson et al., 2023; Lynn et al., 2021; Perl et al., 2021).

2) It is not clear how the irreversibility level was calculated. You mention overlapping sliding windows (of 1 second), but you do not mention the overlap. Both quantities must be specified.

We apologize to the reviewer for the confusion. The text has been corrected and extended in order to improve the readability.

The irreversibility level was calculated on the µECoG signal of all the channels. Irreversibility was measured within non-overlapping sliding windows (of 1 second) analysis through the data (Deco, Perl, et al., 2021), creating two different, forward and time-reversed backward, correlation patterns (the difference of which generates the irreversibility matrices). In other words, both forward and backward correlations were generated at every one second time-window, by calculating the shifted Pearson correlation in the corresponding direction of the signal.

3) It is not clear what the time series x(t) and y(t) are in your example. Two signals from two

electrodes?

We appreciate the comment of the reviewer. We have rephrased the sentence in order to make it more explicit.

The assumed causal dependency between the time series $x\left( t \right)$ and $y\left( t \right)$ (two signals obtained from two different electrodes) is measured through the time-shifted correlation using a sampling rate (fs) of 100 Hz.

4) When you mention detailed balance, you seem to not clearly state what this condition means. For example, for a Markov chain, it is not that the probability of observing a transition between any given two states in the configuration space is the same as the probability of observing the reverse transition. The detailed balance condition is a series of equations that require that that the transition probability matrix P for the Markov process that possess a stationary distribution π satisfies that

πiPij = πjPji for all i, j.

We thank the reviewer for pointing out this ambiguity in the notation. We now have updated the “entropy production” section and changed the notation to differentiate the joint transition probabilities to the conditional transition probabilities often used in Markov chain formalisms

Entropy production is a central concept of nonequilibrium statistical mechanics used to quantify the extent of this violation (Esposito, 2012; Roldán & Parrondo, 2010). To define this quantity, we first consider a system with a set of joint transition probabilities $P\left( i\to j \right) \equiv P\left( x_{t}=i, x_{t+1}=j \right)$. We note that $P\left( i\to j \right)$ differs from the conditional transition probability $P\left( j \right| i)=P\left( x_{t+1}=j |x_{t}=i \right).$In particular, for a Markovian system with a stationary distribution $\pi$, both quantities are related by $P\left( i\to j \right)= P\left( j \right| i) \pi(i)$

5) Your equation 4 is wrong if you consider forward and backward transition probabilities. It

is only correct if you consider joint probabilities of two consecutive time steps, i.e., Pxy =

πxPx→y

As mentioned in the previous answer, we have now changed the notation to clarify this issue.

6) You mention "if the system follows detailed balance" I would say satisfies the detailed balance conditions...the transition probabilities between any two states are equal to the reverse probabilities...again this is wrong... and then the entropy production will be zero.. for this you have to assume that 0 log 0 = 0, which is not explicitly stated in the equations.

We appreciate the comment of the reviewer. We have changed “follows detailed balance” for “satisfies the detailed balance conditions”. Also, we adjusted the text for the new notation and clarified the example of the balanced joint transition probabilities.

If the system satisfies the detailed balance condition, the joint transition probabilities between any two states are equal to the reverse probabilities, $P\left( i\to j \right)$ $=$ $P\left( j\to i \right)$, making all arguments of the logarithms in (6) equal to 1, and consequently leading to null entropy production.

7) In the section results, you mention "We inspected the temporal asymmetry of the dynamical

system", which dynamical system? seems to me that you only look at the experimental time

series without referring to any dynamical system.

We appreciate the comment of the reviewer. We have modified the sentence to avoid possible misunderstandings.

Benefitting from the ferrets’ shorter duration of states, we inspected the temporal asymmetry of the data by extracting both the forward time series and its reverse version.

8) Then you mention that you measure non-reversibility and consequently the level of nonequilibrium of the system. While for some non-equilibrium steady states found in statistical mechanics, this relationship holds, it is not clear to me what you mean here. What is the equilibrium state from which you measure the level of non-equilibrium of the system?

We thank the reviewer for this comment. In this framework we follow previous literature relating violations of the detailed balance to non-reversibility in living systems (Gnesotto 2018, Lynn et al 2021). We corrected the paragraph, clarifying that we do not measure directly the level of non-equilibrium but associate it with the non-reversibility.

By comparing the difference between these two, we were able to extract the level of non-reversibility of the system. Along these lines, irreversible macroscopic dynamics have been treated as a signature of non-equilibrium processes occurring at the micro-scale in biological systems (Gnesotto et al., 2018; Lynn et al., 2021).

9) In the results you mention "If the relation between the network states was the same in both directions (symmetrical), then the entropy level would be 0." Again you are confusing entropy with entropy production.

We thank the reviewer for noticing this confusion. We have updated the statement in order to avoid misunderstandings.

If the relation between the network states was the same in both directions (symmetrical), then the entropy production level would be 0.

10) Fig 3f, you show transition probabilities but the values that you show reveal that these are not transition probabilities, as the sum of each row is not one.

We thank the reviewer for noticing this detail. The displayed matrix is the joint transition probability, which sum is normalized to 1 without normalization of the rows. We have changed the caption to:

(F) The joint transition probability between the network states is displayed for each behavioral state. Furthermore, the difference matrix (upper triangle minus lower triangle) is displayed to enhance the visualization of the effect

11) In the discussion you mention "We show how this measure of irreversibility can be used as a proxy of non-equilibrium", Actually you don’t show that, you only vaguely mention it.

We thank the reviewer for the comment. We have updated the statement in order to clarify the idea behind it.

We measured the irreversibility of the data and implemented it as a proxy of the degree of non-equilibrium. Furthermore, this approach can be used in a manner to localize the differential influences of various cortical subsystems on this phenomenon.

12) In the discussion you mention "Previous studies have inspected non-equilibrium of signals in macaque monkeys (Deco et al. 2022)" You forgot one relevant article by one of the authors of this manuscript (Sanz Perl 2021).

We apologize to the reviewer for this omission. We have added the mentioned reference in the corresponding section

Previous studies have inspected non-equilibrium of signals in macaque monkeys (Deco et al., 2022; Perl et al., 2021), allowing to disentangle different consciousness levels through the non-reversibility of the signal.

13) In the discussion you mention that the SWS behavioral state exhibited a lower entropy production, with respect to the awake state. This result is in agreement with the recent result obtained using human fMRI data (Gilson 2023), a relevant relationship may appear here, you should compare and comment on this.

We thank the reviewer for the comment. We have incorporated the suggested article and a add a sentence relating our results.

Previous studies have found distinctive levels of entropy production at different awareness states through fMRI data (G-Guzmán et al., 2023; Gilson et al., 2023) showing convergent evidence with the results presented in this study, disregarding of the used neuroimaging technique.

14) In the discussion you mention " In conclusion, we demonstrated how to direct metrics derived from thermodynamics such as signal reversibility". Your custom measure of signal reversibility (equation 3) seems not to be derived from thermodynamics.

We corrected the paragraph, clarifying that we do not directly measure the level of non-equilibrium but associate it with the non-reversibility.

In conclusion, we demonstrated how metrics related to thermodynamics such as signal reversibility, which have been treated as a signature of non-equilibrium processes, reflect brain dynamics distinguishable between different behavioral states.

***Minor :***

1) In the definition of determinism you mention "The average determinism of a directed network X with N .. with N what?

We thank the reviewer for pointing out this omission. The explanation has been expanded in order to be clearer.

The average determinism of a directed network X with N (amount of states in the transition probability matrix) can be quantified as

2) In the definition of degeneracy, it is not clear to me how you compute the entropy of an average. Is the distribution of averages? Also, seems that a parenthesis is not necessary and you define a network as X and then refer to it as x.

We thank the reviewer for the suggestions. We have updated the formulas getting rid of the unnecessary parenthesis and replaced the X with the corresponding lower-case symbol. Furthermore, we added an extra paragraph to clarify how degeneracy was calculated.

The average determinism of a directed network $x$ with N (amount of states in the transition probability matrix) can be quantified as

$$Det\left( x \right)= \frac{{log}_{2}N- <H(W_{i}^{out})>}{{log}_{2}N}$$

Degeneracy is calculated as

$$Deg\left( x \right)= \frac{{log}_{2}N- H<(W_{i}^{out})>}{{log}_{2}N}$$

Where the $\left( W_{i}^{\mathrm{out}} \right)$ is a vector in which every component contains the mean probability of transition of each state. Therefore, the entropy is calculated over the distribution defined by the components of the vector.

3) In the definition of mutual information, the definition itself is missing, you only mention properties and mention "strictly independent" while being independent is a property that a pair or random variables holds or not. Statistical interdependence is different.

We thank the reviewer for the comment. We have re-ordered the Mutual information section in order to enhance clarity while adding the corresponding formula.

Therefore, as a result, mutual information is calculated by adding the information (entropy) of the previous (H(X)) and future (H(Y)) state and subtracting the joint entropy (H(X,Y)) of them. The equation is as follows:

$$MI\left( X;Y \right)= H\left( X \right)+H\left( Y \right)-H(X,Y)$$

(10)

4) In the results you mention "The absolute difference between the irreversibility matrices (forward minus backward)" what do you mean by irreversibility matrices, seems to me that you compute the difference between the functional connectivity matrices.

We appreciate the reviewer for discussing this concept. In the methods section, we explained how the irreversibility matrices are calculated:

For the forward evolution the time-shifted correlation is given by

$C_{forward}\left( \Delta t \right)=<x\left( t \right),y(t+\Delta t)>$	(1)

And for the reversed backward evolution the time-shifted correlation is given by

$C_{reversal}\left( \Delta t \right)=<x^{(r)}\left( t \right),y^{r}(t+\Delta t)>$	(2)

Therefore, the absolute difference between these two matrices provides the irreversibility value

5) Fig 1c what is the time scale? After you mention that you compute irreversibility values (difference between forward and backward matrix) at each time point. It is not clear at all how this quantity is computed following your definition in Equation 3. Is an average over all pairs?

We thank the reviewer for the comment. We have clarified the caption of Figure 1c in order to enhance clarity:

Frequency spectra, classification of behavioral states and irreversibility values (difference between forward and backward matrix) at each time point across the whole recording (> 3 hours).

Regarding the irreversibility calculation, indeed it is an average over all pairs. The explanation is provided in the corresponding methods section above equation 4:

The pairwise level of non-reversibility is given consequently by the absolute difference between the assumed causal relationship between these two timeseries in the forward and reversed backward evolution, at a given shift $\Delta t=1/ts$. For the current study the shifting was selected at T=1.

$I_{x,y}\left( T \right)= {|C}_{forward}\left( T \right)- C_{reversal}(T)|$

(4)

6) How do you explain the differences between irreversibility and entropy production between panels C and I?

We thank the reviewer for the opportunity to comment on this topic. We have added a paragraph to the result section discussing this issue.

It is relevant to clarify that even if the AA/REM state revealed to be the predominant state in both metrics (irreversibility in the source space and entropy production in the latent space), the order between the remaining two states is inconsistent. Further studies should dive into this difference to understand this phenomenon.

7) In the discussion you mention "the extensive recording time (>3 hours per animal)". This should appear before not in the conclusion. Before you only mentioned resting activity during the light cycle between 12 a.m. and 6 p.m., but is not clear if you used all the data.

We thank the reviewer for the comment. We have incorporated the length of the recording in the methods and the result sections:

Neural activity was recorded over a large portion of the left cerebral hemisphere including visual, auditory, and parietal areas (more than 2 hours recording per animal).

We obtained data from 4 animals in resting activity during the light cycle between 12 a.m. and 6 p.m. (more than 2 hours recording per animal).

8) If you want to illustrate a transition probability a color bar with the values is missing.

We thank the reviewer for the comment. The transition probability matrices display their color bars with their corresponding values as seen in the following image:

9) In figure 1 is not clear what HMM-MAR brings to the irreversibility framework.

We thank the reviewer for pointing out this topic. We have extended the result section where figure 1 is presented to make more explicit the reasoning behind the addition of the HMM-MAR analysis.

We incorporated complementary approaches in order to analyze the embedded dynamics of the system and their differences in the different brain states. With that goal, and to add robustness to the obtained results, we inspected recurring activity patterns across time by applying HMM in order to observe, in a different way, the embedded dynamics in the signal.

10) In the caption of panel I) you mention "The highest entropy" do you mean entropy production?

We thank the reviewer for pointing out this topic. We have updated the figure caption to “entropy production” to avoid misunderstandings.

11) Figure 2B is badly explained. Took me a while to understand what you did and I’m not sure I understood. You should be more explicit on what each column means. Only the color of the rectangle guides what you mean. Also, you should be more explicit in the x-axis of the histograms. It is not clear how you measure irreversibility between and within regions

We thank the reviewer for the comment. We have improved the figure by adding a title to each column and the x-axis of the bar plot:

Furthermore, we expanded the results explanation to enhance the interpretation of the within and between regions calculations:

By means of paired t-student comparisons, we found a significant difference between the within-regions (connections between brain areas of the same regions) and between-regions (connections between brain areas of the different regions) irreversibility values (p<.01).

12) In my opinion, the computational code used to obtain the results should always be available, readable, and usable without even needing to request it.

We agree with the reviewer as we have shared the code used in our GitHub repository. A sentence providing the link was added in the methods section:

Code and sample data are available at: https://github.com/SebastianIdesis/Hierarchy_Ferrets-2023-
